# Notes of an European Tour

**Published:** 1847-05

**Authors:** F. H. Hamilton


### BUFFALO MEDICAL JOURNAL

AND

### MONTHLY REVIEW.

**VOL. 2.** MAY, 1847. **NO. 12**

### ORIGINAL COMMUNICATIONS.

ART. I.—*Notes of an European Tour. Concluding letter.* By F. H. Hamilton, M. D.

Holland is a very proper sliding off place, to one who has been travelling over the continent of Europe. It is a sort of "land's end," where by a gradual declination you wear off into the sea.—Holland is in fact nothing more than a hollow lodgement in the bed of the ocean, secured by a vast coffer dam, or as Butler says,

> "A country that draws fifty feet of water,
> In which men live as in the hold of nature;
> And when the sea does in upon them break,
> And drown a province, does but spring a leak."

And this strange country is not only surrounded with water, and sunk in water, but it is every where intersected with water; rivers and canals being the almost universal substitutes for roads, streets and even fences. To speak of a Dutch landscape in an awkward solecism; yet if I was asked what constituted the most prominent features in a Dutch landscape, I would answer water and wind-mills!

On the right bank of the Maas, 24 miles from the sea, was once, many centuries past, a marsh of deep quagmire, into which the industrious Dutchmen drove long spiles like masts, brought with incredible expense and labour from Norway; and upon the top of these Norwegian forests they built a city, now called Rotterdam.

It is a quaint old town, with as many canals as streets ; with ferries and drawbridges, and with clumsy ships sailing familiarly between its side walks ; with brick houses whose gable ends always peer out upon you so inquisitively ; without dirt or any such thing, but abounding in herring. Indeed it is filled with quaintness and neatness and fat contentment. Next to my hotel on the *boompjes* is a drug store, and over the door is placed the wooden bust of a Turk, wearing a turban, and with his mouth stretched painfully wide. It is called a "gaper," and is the usual sign of a drug shop. * *

Through Delft and the Hague I came to Leyden, a place associated with the memory of many of the most distinguished men in our profession. Here was the great University which, established in 1575, soon attained such eminence as to gain for Leyden the appellation of the "Athens of the West." Its medical department especially, was during the 17th and 18th centuries recognised as the first in Europe ; and its pupils became so numerous, that it is said, sufficient lodgings could not be procured within the town. A few of its distinguished names are worthy of being recalled.

Francis Sylvius was made professor of Medicine at Leyden in 1658, and early laboured to convince his pupils of the corectness of the recent discoveries of Harvey in relation to the circulation of the blood ; but while he was thus doing much for the advance of medical science, he contributed no little to retard its progress by a fanciful theory which he held, and to which he gained many disciples, that all diseases consisted in certain chemical changes of the blood ; either an excess of alkali or of acid. He died in 1672. He is not to be confounded with James Sylvius who lectured in Paris, and who has left his name attached to a small fissure in the temporal bone.

Pitcairn, one of the principal leaders of the mathematical sect, and whose lectures were published in a work entitled "Elementa Medicinæ Physico-Mathematica," was made professor of physic in 1692 ; but his doctrines not being favourably received, he relinquished his place and returned to his native city, Edinburgh.

Bidloo was born at Amsterdam, and in 1694, having already greatly distinguished himself as a surgeon, he was called to the chair of anatomy and surgery at Leyden. He published more than one hundred anatomical plates, which are in many points inacurate, yet they give us all the knowledge which was at that time possessed upon this subject.

Walther, who wrote a dissertation "De Lingua Humana Libellus," succeeded to the same chair in 1723, and to Walther succeeded Albinus. Albinus was appointed when only 20 years old, and he continued to lecture upon the same branches for more than half a century.

But the university is mostly distinguished as being the field of the labours of the immortal Boerhaave, who was chosen professor of the Theory of Medicine in 1701, when he was 33 years old; after which he successively occupied the chair of Practice of Medicine, Chemistry and Botany, and was twice made Rector of the university. By his large practice, his lectures and especially by his exceedingly parsimonious habits he acquired great wealth, and was thus enabled to purchase for himself a beautiful country seat near Leyden, which is still standing, much in the same condition as he left it. It is surrounded by little green canals, and the grounds are laid out with great regularity and neatness, if not as much taste.—To this place he retired with his wife and daughter in 1720, having become too old and corpulent to continue with his former zeal his studies and practice. He died a few years after with a disease of the chest, and was buried in the old church of St. Peter's at Leyden. Upon his monumental stone, erected by the citizens, is the following inscription: "Salutifero Boerhaavii Genio Sacrum."

Entering the university buildings, to which Boerhaave and his colleagues for so long a time attracted such crowds of listeners, one is astonished to find that they are quite small and ordinary in their appearance and accommodations, and when in connection with this we call to mind the retired situation of the city and the smallness of its population, we are compelled at once to believe that it was the reputation of its teachers alone which acquired for this school so great a name.

But after the withdrawal of Boerhaave, its reputation began rapidly to decline, and at present it educates only a few medical students from its own province. There is however connected with it a very excellent museum of comparative anatomy, and a choice and well arranged botanical garden under the care of Prof. Reinwardt. The garden is the same in which Boerhaave loved so much to walk and converse upon the beauties of nature as seen in the growth and developement of plants, and where, dressed in the plainest style, like a common gardener, he was often found with the spade and hoe. The large flowering ash (fraxinus ornus) growing in one side of the garden, is said to have been planted by Boerhaave.

Of Harlaem and its prodigious organ—of Amsterdam and its 200,000 inhabitants, living, like the people of Rotterdam, on the tops of trees, I have little to say.

The Dutch provide well for their sick and infirm, and Amsterdam alone has twenty-three public charities. These are supported by a tax upon all places of public amusement and by voluntary contributions. Lest you might forget the poor, for you seldom see a beggar in Holland, you are constantly reminded of their existence and necessities by "contribution boxes" in the churches, in the houses and in the streets even. It is said that when Louis 14th meditated an attack upon this city, Charles the 2d who had lived among them during a considerable portion of his exile, remarked, "I am of opinion that Providence will preserve Amsterdam, if it were only for the great charity they have for the poor."

You ought also to know that Amsterdam has the honor of being the first city which formed an association for the purpose of recovering persons drowned. This association was established by a number of wealthy gentlemen in 1767, and in four years they had rescued between twenty and thirty. It seems very natural that a city situated like Amsterdam should have taken the lead in this kind of charity.

---

Dr. Flint—I had designed to continue my notes through England, Scotland and Ireland, many of the cities of which I visited during my absence, and to have spoken of the numerous hospitals and the many distinguished physicians and surgeons with which Great Britain is adorned ; to some of whom I feel under great and lasting obligations for the many civilities which they extended to me. But I am admonished by the length to which these letters have already been extended that I ought to spare your readers from farther inflictions, and to close up my pencilings in the homely couplet of the rhyming cobler.

"Songs thirty have I sung, yet ten remain.
Crude, undigested, written in the brain."

I am the more especially induced to leave you here, because so much time has now elapsed since I was in Great Britain, that much that I would write can no longer be new, and therefore scarcely interesting, while upon all that pertains to the continent, where things are less mutable, or rather indeed where it is only the old that possesses interest, time brings fewer changes, and not much that is new, save from a few points, is worth recording.

I opened my portifolio originally with the intention of giving you a few of the hastily written letters which were addressed to a much esteemed professional friend at home, but I have been finally carried considerably beyond this first design, and have enlarged my letters by a very copious draft from my notes. In closing this I have pleasantly refreshed my memory upon scenes and circumstances of my travel which to me, possessed at the time an intense and almost indefinable interest. These impressions I was willing to fasten before they had altogether faded from my mind. In this alone I think myself compensated for my labour, but if I have in any degree helped to entertain or instruct the readers of your most valuable journal, I am so much the more rewarded.

Yours truly, F. H. HAMILTON.

